# A Case Report of Candidiasis Cellulitis in Long-Term Corticosteroid Use

**DOI:** 10.1155/carm/6140044

**Published:** 2025-09-30

**Authors:** Minoo Heidari Almasi, Afsaneh Safarian, Seyyed Amirhossein Salehi, Hamideh Moradi Shahrebabak

**Affiliations:** ^1^Imam Hossein Hospital Clinical Research Development Unit, Shahid Beheshti University of Medical Sciences, Tehran, Iran; ^2^Department of Internal Medicine, Loghman Hakim Hospital, Shahid Beheshti University of Medical Sciences, Tehran, Iran; ^3^Student Research Committee, School of Medicine, Shahid Beheshti University of Medical Sciences, Tehran, Iran

**Keywords:** abscess, betamethasone, *Candida albicans*, case report, cellulitis, corticosteroid, fluconazole, fungal infection, immunosuppression, opportunistic infection

## Abstract

Fungal infections are a significant global health concern, resulting in over 1.5 million deaths annually. Among these, *Candida albicans* remains a prominent pathogen responsible for various cutaneous conditions, despite its typical role as a benign commensal organism. This case report details a 54-year-old male patient with a cutaneous abscess attributed to *C. albicans*, likely exacerbated by prolonged betamethasone injections for respiratory issues stemming from chemical exposure during the Iran–Iraq War. The patient presented with a month-long history of progressive hand swelling and pain, exacerbated despite antibiotic treatment. Clinical examination and imaging revealed a significant fluid collection in the affected wrist, prompting surgical drainage. Subsequent culture confirmed the presence of *C. albicans*, while blood cultures remained negative, underscoring the localized nature of the infection. This report highlights the opportunistic potential of *C. albicans*, particularly in immunocompromised individuals, and emphasizes the importance of considering fungal infections in patients with prolonged corticosteroid use. The patient responded favorably to oral fluconazole, with complete resolution of symptoms by follow-up. This case illustrates the need for awareness and prompt identification of fungal infections in at-risk populations, advocating for a more comprehensive approach to patient management in similar clinical scenarios.

## 1. Introduction

Fungal infections represent a critical global health concern, with over 1.5 million individuals losing their lives each year, a mortality rate comparable to that of *tuberculosis*. The most prominent fungal pathogens associated with severe disease include *Candida* spp., *Cryptococcus* spp., *Pneumocystis jirovecii*, and *Mucormycetes* [1]. Among these, *Candida albicans* stands out as the leading cause of common fungal infections. This yeast typically colonizes various mucosal surfaces and skin without causing harm; however, under certain conditions, it can become pathogenic, leading to a spectrum of infections. Cellulitis is defined as an acute bacterial or fungal infection of the dermis and subcutaneous tissues, characterized by erythema, swelling, warmth, and pain, which can progress to complications such as abscess formation if untreated (include the definition of cellulitis in the introduction) [2]. Cutaneous candidiasis presents with a wide array of clinical manifestations, including chronic and acute paronychia, onychomycosis, Erosio Interdigitalis Blastomycetica (EIB), folliculitis, diaper dermatitis, vulvovaginitis, and balanitis. A frequent skin manifestation of this fungus is the involvement of the *epidermis*, which can progress to cellulitis [3].

A substantial portion of the global population receives systemic glucocorticoid therapy to manage a variety of conditions, including respiratory diseases (such as asthma), rheumatic disorders (such as giant cell arteritis and rheumatoid arthritis), and certain neoplastic conditions. Prolonged use of corticosteroids is a significant risk factor for opportunistic infections [4]. Those with compromised immune systems, such as individuals suffering from uncontrolled diabetes, HIV infection, or those undergoing organ transplants and immunosuppressive treatments, are especially vulnerable. These individuals face an increased risk of developing serious and potentially life-threatening mucocutaneous infections caused by *Candida* species [5].

This case report presents a patient with a cutaneous abscess caused by *C. albicans*, occurring in the absence of fungemia and potentially associated with prolonged betamethasone injections. This highlights the need for awareness of fungal infections in patients receiving immunosuppressive therapies, as timely diagnosis and management are crucial for positive outcomes.

## 2. Case History and Examination

This case is presented to you, after full written consent was given to the writers by the patient. A 54-year-old man came to our department with gradually worsening redness and swelling of his left hand. He was married, lived in Tehran, and was an employee of a bank. He mentioned that the pain, swelling, and redness of his hand started a month ago. During this time, he had visited a general practitioner several times and had been treated with oral antibiotics but had not recovered. He had also been experiencing persistent difficulty in breathing due to previous chemical exposure during the Iran–Iraq War 30 years ago, and he used injectable betamethasone without a doctor's prescription monthly at a dose of 8 mg intramuscularly since more than 15 years ago (For how long was betamethasone administered?).

**Figure 1:**
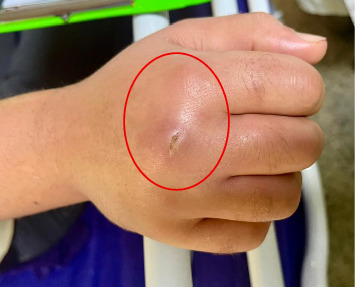
Photograph of the patient's left hand showing swelling and redness caused by a cutaneous abscess due to *Candida albicans* (it is recommended that the microorganism be indicated on the label in Figure 1 and the lesion be highlighted in the photograph). The lesion is highlighted to emphasize the area of infection, characterized by fluctuant swelling and erythema extending to the wrist.

During the examination, the patient did not have a fever, and his vital signs were stable. We observed that his left hand was swollen to the top of his wrist, warm to the touch, and fluctuant, accompanied by redness. Despite this, the flexion of his fingers was painful and limited. The patient exhibited normal radial and ulnar pulses. Other examinations were normal (Figure 1).

### 2.1. Differential Diagnoses, Investigations, and Treatments

After he was hospitalized, routine lab tests were requested in the emergency room. We ordered a wrist X-ray and a soft tissue sonography based on his description. The ultrasound revealed normal venous flow but showed evidence of fat stranding and pockets of fluid accumulation in the soft tissues of the wrist and the distal part of the forearm. The measured dimensions of the collection were approximately 44 by 28 by 14 mm, containing around 8 cubic centimeters of fluid. In lab tests, the complete blood count (CBC) showed leukocytosis of 13,000, with 80% neutrophils, and increased erythrocyte sedimentation rate (ESR) and C-reactive protein (CRP). Electrolytes, renal function tests (including creatinine and blood urea nitrogen), liver function tests (including AST, ALT, ALP, and bilirubin), and coagulation profile (INR and aPTT) were all within normal limits. Blood glucose levels were also normal, ruling out hyperglycemia as a contributing factor to infection.

Subsequently, the decision was made to transfer the patient to the operating room for direct drainage of the contents of the abscess, which were removed, and the area was thoroughly cleansed. Samples were collected and sent to the laboratory for culture and smear. After submitting the abscess sample, intravenous vancomycin was started at 1 g every 12 h for the patient.

### 2.2. Outcome and Follow-Up

**Figure 2:**
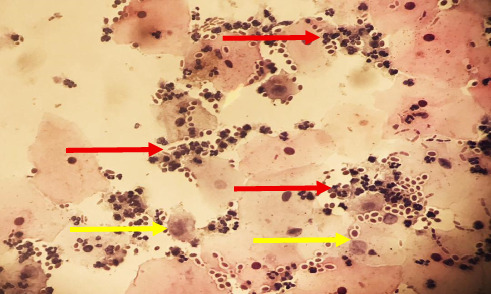
Histology image of the abscess smear showing *Candida albicans* yeast cells and damaged tissue cells (H&E staining). The evaluated structures, including budding yeast cells (red arrows) and areas of tissue damage (yellow arrows), are marked with annotations to highlight their presence and distribution (• In the histology, it is recommended that the area where the evaluated structures [yeast and damaged cells] be marked).

The direct smear of the abscess displayed budding cells, while the abscess culture confirmed the presence of *C. albicans* (Figure 2). Additionally, the mycology analysis revealed the isolation of colonies of *Candida* species. The patient's blood cultures were negative on two occasions with an interval of 12 h. Although the blood cultures were negative, a transthoracic echocardiography was performed. With the exception of a slight increase in pulmonary artery pressure, other findings were normal, and no evidence of vegetation was found.

Due to the culture results of the abscess sample, intravenous vancomycin was discontinued, and oral fluconazole was started at 200 mg every 12 h. After 48 h of oral antifungal treatment, erythema, swelling, and warmth of the patient's hand decreased, and the patient was discharged with oral fluconazole. After 2 weeks, the patient was seen on an outpatient basis, and his hand cellulitis was completely healed.

## 3. Discussion

*C. albicans* is a versatile fungus that forms part of the human microbiome. Typically, it coexists harmlessly with most individuals as a lifelong commensal organism. However, certain conditions can cause *C. albicans* to lead to a range of infections, which may vary from minor skin issues to severe, life-threatening systemic infections [6]. While abscesses are rarely attributed to *C. albicans*, the yeast is more commonly associated with dermatitis and mucous membrane infections [7].

Individuals with compromised immune systems are at a heightened risk of developing infections caused by *C. albicans*. This vulnerable population includes premature neonates, individuals with diabetes, HIV-positive patients, and those undergoing organ transplants or chemotherapy [8].

In the case we reported, the extended administration of betamethasone injections to the patient, in conjunction with a prior history of chemical exposure that resulted in respiratory complications, likely contributed to the compromise of his immune system. This weakened immune state could have facilitated the emergence of a localized cutaneous infection induced by *C. albicans*, highlighting the pathogen's opportunistic nature in individuals at risk. This case underscores the critical importance of considering fungal etiologies, particularly in populations with compromised immune defenses.

Corticosteroids are widely used for their anti-inflammatory and immunosuppressive properties, but their prolonged use can significantly compromise the immune system, leading to an increased risk of opportunistic infections, including those caused by *Candida* species [9]. These medications work by inhibiting the production of proinflammatory cytokines and reducing the activity of immune cells, such as T lymphocytes and macrophages, which play crucial roles in fighting fungal infections. As a result, patients on long-term corticosteroid therapy experience diminished phagocytosis and impaired immune response, allowing *Candida* organisms, which are normally kept in check by the host's immune defenses, to proliferate [10]. This immune suppression creates an environment conducive to the development of localized infections, such as cellulitis, as seen in patients with a history of corticosteroid use. There has been a report of *Candida* cellulitis following local corticosteroid injection [11]. Understanding the mechanisms of immune suppression associated with corticosteroid therapy is essential for healthcare providers to identify at-risk patients and implement appropriate preventive and therapeutic measures.

There have been prior reports of *C. albicans* causing cellulitis in different parts of body. Om-Sub Kwak et al. reported the case of a 50-year-old woman with diabetes presented with persistent redness and swelling in her cheeks, initially treated with incision and drainage and antibiotics, but showing no improvement. A computed tomography (CT) scan revealed diffuse cellulitis and chronic maxillary sinusitis. Pus culture identified *C. albicans*, leading to intravenous amphotericin B treatment. Additional cultures revealed *Acinetobacter baumannii* and coagulase-negative *Staphylococcus*, along with a confirmed aspergilloma. After 1 week of treatment, her condition improved, and she was discharged on oral fluconazole for 3 weeks. She remained recurrence-free during a 4-month follow-up period [12].

Something that could potentially be worrisome is the occurrence of *Candida* cellulitis caused by nonalbicans *Candida*. In a recent case report by Tolaj et al., it is stated that a 52-year-old male was admitted with erythema, edema, and tenderness in the submandibular region that failed to improve with outpatient antibiotics. His CT scan revealed tissue edema, worsening his condition. An incision was made, and pus culture identified *Candida guilliermondi*. He was started on intravenous fluconazole, leading to significant improvement within 3 days. The patient was discharged after 7 days on oral fluconazole for 1 week, with no recurrence of cellulitis noted at a 4-week follow-up [13].

The literature on *Candida* cellulitis remains sparse, primarily consisting of isolated case reports that do not fully capture the complexity of this condition. This limited body of work indicates a significant need for a more comprehensive compilation of data regarding the diagnosis and management of *Candida* cellulitis across diverse patient populations.

A focused effort to gather, analyze, and synthesize findings from various clinical cases could greatly enhance our understanding of this rare yet significant infection. Such an endeavor would not only provide insights into the clinical presentation, risk factors, and effective treatment options but also facilitate the development of standardized guidelines for managing *Candida* cellulitis. Perhaps designing a systematic review and meta-analysis study would be the answer to this need.

### 3.1. Key Clinical Message

Prolonged corticosteroid use increases the risk of opportunistic fungal infections such as *C. albicans* cellulitis. In immunocompromised patients with nonresolving cellulitis despite antibiotics, consider fungal etiology. Prompt culture-guided diagnosis and antifungal therapy (e.g., fluconazole) ensure resolution. Heightened clinical suspicion in high-risk populations is critical to avoid delays in treatment.

## 4. Conclusion

In conclusion, this case highlights the significant risk of *C. albicans* infections in patients with compromised immune systems, especially those receiving prolonged glucocorticoid therapy, such as betamethasone. Despite being a part of the normal microbiome, *C. albicans* can become pathogenic under certain conditions, leading to serious infections that can manifest as cutaneous abscesses, cellulitis, or other mucocutaneous complications. The patient's response to oral fluconazole after drainage and culture confirmation emphasizes the importance of recognizing and treating fungal infections in vulnerable populations. Given the rising incidence of fungal infections worldwide and their potential lethality, healthcare providers must maintain a high index of suspicion for opportunistic pathogens such as *C. albicans* in immunocompromised individuals. Early diagnosis and targeted treatment are crucial for improving outcomes, thus advocating for increased awareness and prompt management of such infections in clinical practice.
